# Supplementary material for: Nanog regulates Pou3f1 expression at the exit from pluripotency during gastrulation
Source: Biol Open. 2019 Dec 2;8(11):bio046367. doi: 10.1242/bio.046367 (PMC6899006; doi:10.1242/bio.046367)
Supplement: Supplementary information [file biolopen-8-046367-s1.pdf]

Supplementary Figures

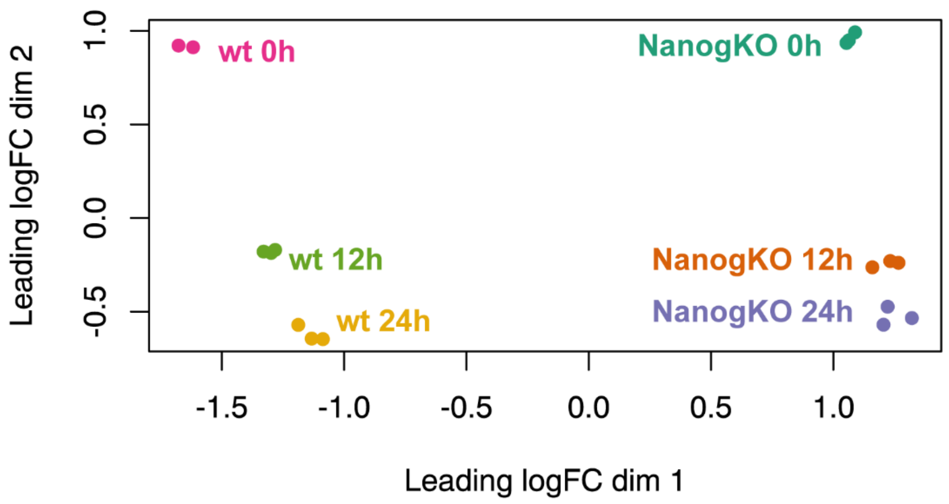

Fig. S1: Principal Component Analysis of the RNA-seq samples of early naïve to primed differentiation of Nanog KO and control ES cells. Component 1 (horizontal axis, 43% of variability explained) separates samples according to their genotype, while component 2 (vertical axis, 26% of variability explained) separates samples by time of differentiation (from 0h to 24h).

Biology Open • Supplementary information

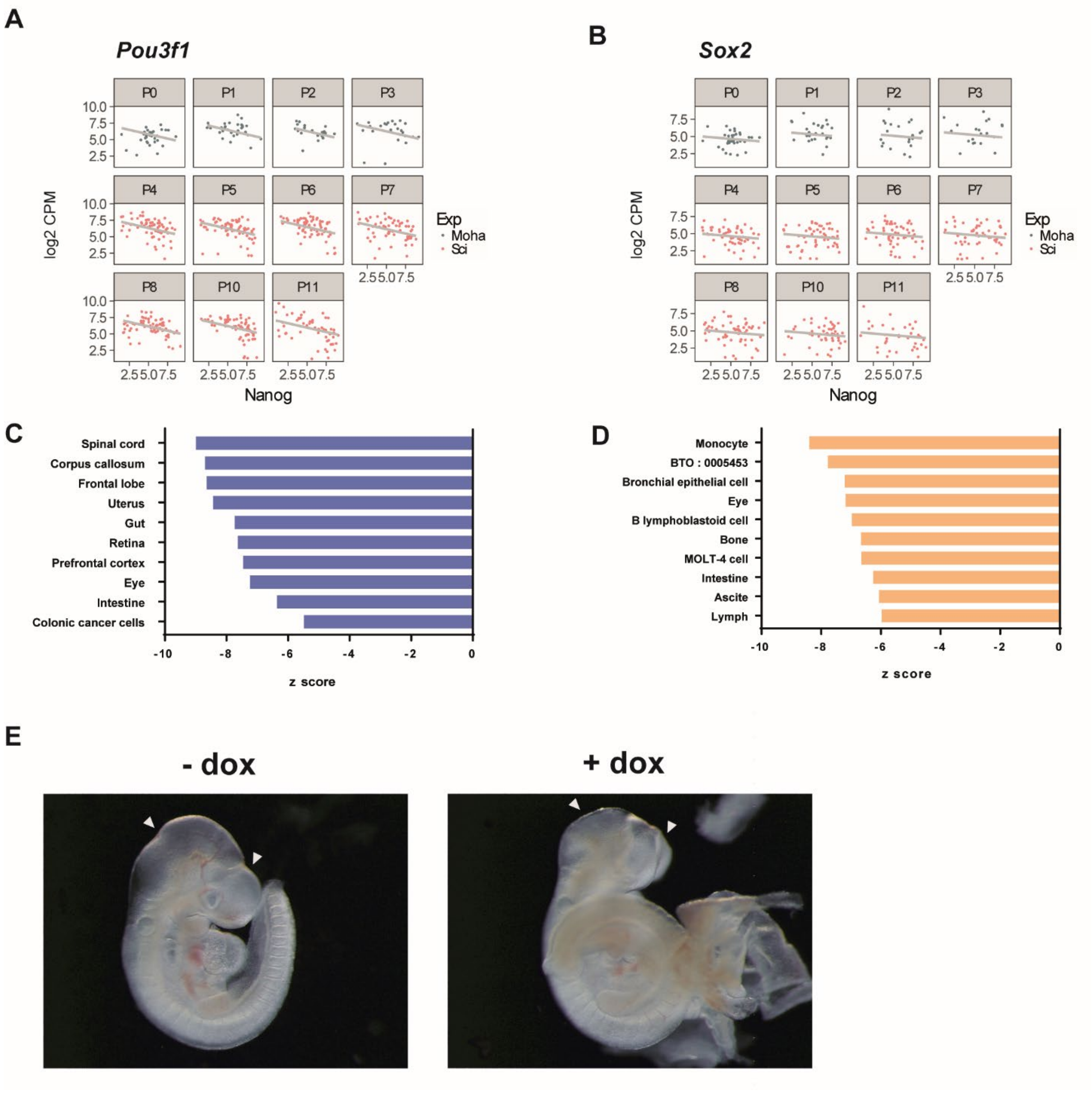

Fig. S2. Combined analysis of the RNA-seq datasets. (A, B) Graphic representation of the correlation in separate single cell E6.5 embryo RNA-seq samples (P0 to P11) from Mohammed et al (2017), in blue, and Scialdone et al. (2016), in red, of *Nanog* with *Pou3f1* (A) and *Sox2* (B). Expression values are expressed in Log2CPM. (C, D) Enrichr results of Jansen Tissue Gene set library for genes whose expression is negatively (C) or positively (D) correlated with *Nanog* in E6.5 single cell RNA-seq datasets. (E) Bright field images of freshly dissected E9.5 *Nanog*^{tg} embryos treated (+dox) or untreated (-dox) with doxycycline from E6.5. White arrows indicate the craniofacial defects observed upon *Nanog* expression. Scale bar, 500μm.

Biology Open • Supplementary information

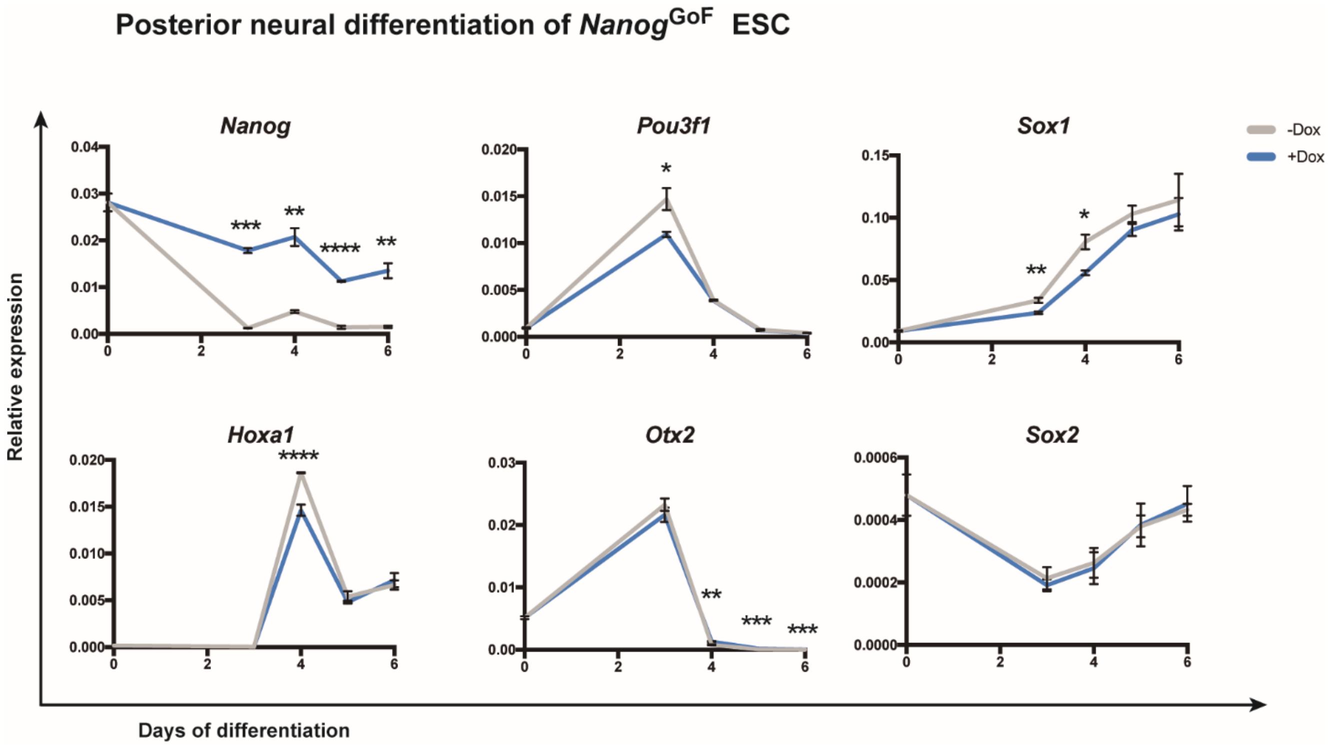

Fig. S3. Expression of selected markers during posterior neural differentiation of ES cells. Expression of *Nanog* and neural markers, as quantified by RT-qPCR, during 6 days of differentiation to posterior neural fate of *Nanog*^{tg} ES cells with (+dox, blue) or without (-dox, gray) doxycycline. n=3 at each time point; *, p<0.01; **, p<0.001; ***, p<0.0001, ****,p<0.00001, by student's t-test.

Biology Open • Supplementary information

A

-9Kb locus

AGGACCCCGCAGCGTCCCCATCTAAGCTAAGTCAGGATCTATATTCCCTG
GGAAAGGGTCTGGCCTGTGCTGGAACAGTCATCTCCACTAGATTTAGCCT
GCCCACTACAGAGGCTAGTATGCCCGGGTTTGTGGATGTCCCTGGCCTTC
CCTGATTTTCCCTGCTCCTACTGGCACGAATGATTTCCTCTCCTACCATT
CTATTCATCTTCATTTTGGTCGTGGATCAAGGCTATTAGGCAAAACTGAG
CCCT AATAGGCCCCCCTCCTAGTGCTCTGGGTCCATTAACCAGCCCACTT
TGACGACAAGCAATGACCCAGTGTGAATAAGAAGATCTACCCGCTTCTCT
GAAGCGGAAAGCCAGAAACGGCAATATCCTCTCGTCATCCCCCTTTTCAA
AAGAAAGCACTAAGCCAGGCATGGTGGCGCACACCTGCAATCCCAGAACC
CAGGAAGCTGAGGCAGAAGAGCCAGAAGGACCATTGATTCCAAGCCAGCC
TGATTTACATAGCAAGAGCCGGTCGCCAAAAGCTAAGGAAATAAATAAAA
AAACTTAAAAGAGGAAAGTAAGTGGGCACACCTTTAATCCCAGTATTGGA

-9Kb PCR gel

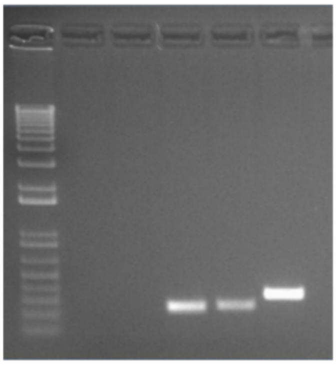

373bp wt
243bp del

B

-11.5Kb locus

TGCAGGGCCCTGAAGCCTGACAGAAAAATCACAGACACACTCTGACATTA
ATCAAAGACTCTCATAAATAAATATGTGTAAATCATATGCAAACCAGGTA
GGAATTTACATCTCTCTCTCTCCCCCTGATGAGTCAGCACCTCCAGACAC
CAGGGAGGCTGCCTGCTTGCTGCTCTCTCGGGAGGTTTAGGACAAGAGCC
CTCAACATCCAAGGGAGCCCGCTGAATTCACGATCTCAGATCCTAAATGT
CCATGCCATCAATATAAATCATGCAATTTGCCACAATGCTTTCTCCTACA
CACCAACTACTAAAAGCGTTTAATGAGAAACTATGTGAAGCTCTCAGAAG
GGTCGTTGGTGGTATGCACACAGCTTACCTACACTCTTCTTCATATTATC
TCATCTAGCTTTCACAATAATAGCAATACTCATTACAACAATCATAAAAG
TAATAGGTGTTCTTCTGTCGTGGAGGCTTCCACGACGTCCCTGTGTGCGG
CTCCCGAGGTCCAGTCTTCCACCCCCATCCCTCCAGGCCCTACCATTTCA
GTCCACTCCAGCCTGGCTTCCTACTGCCAGGAACTGCAGCTTTGCCTGAA
GGCTTCTGCAGATCTCCTGGAGGCTGCCCTCCCCTCCTAAGGCAGGCCAC
AAGTGTTGGAGAGTCAGCCCCCACAGCAGAGA

-11.5Kb PCR gel

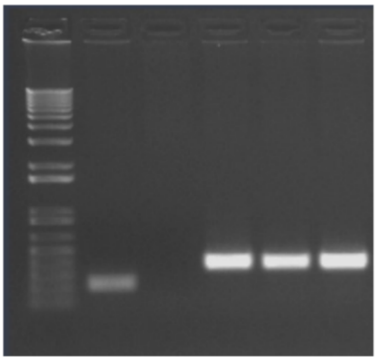

367bp wt
210bp del

C

+9Kb locus

AAGTCTGACTGAAGCTTCCTTCAGGTCTCCGTGAGTAGTTTTACTGCAAC
CCTAGCCAGAGGTTAAGCAGAGGTTAACCAGCCTCAGACATTTGGGGTCA
CCAAATGGATCATTTGTCACCTTGGACCTCAAACTAGGAAGTAACCATTC
CTCCATTCACTTATCCATCAGTCCGTTCTCCAGCCAGCTACTCCTCCATT
CACCGAGCCACCCACCTTTGCATGCAGCCATTCACCTACCTACCTATTCA
GCTACCCACCCATCATCTGCCATCCATTCATCCGATCTTCTTGCTTAACG
AATGTCAAGAAGGCTGATGGCCTCTAACCTCCACGCTCTTCTAGCTTTGT
CTCATTCCCTTCCTGCCCCTCAGCCATATAGGGTGTTTGAGAAAACGGTG
TAGAAGGTAATGATGAAACCTGTTGTCATATCTACGAAGTTGTGTGAGTC
CAAGTGCTGGAACTTTCCACACTCCCCTCCTCTCCCCAAAGAAGCTATAG
TTGATACAGCCTGTGGCAAAAGTCTAGATGGCCTTAGTTCTCATTTCTTA
CCCCAGGAATGTCAAGTTCTGGGCTAAATGTAAGGGGTTTCAAAGGTCAC

+9Kb PCR gel

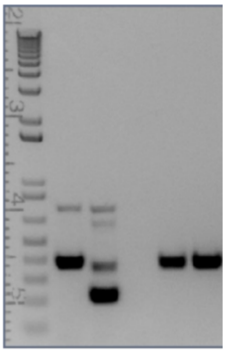

386bp wt
200bp del

PCR PRIMERS **GUIDE RNA**

Fig. S4: Deletion of NANOG bound genomic regions from the *Pou3f1* locus. Sequence (right) of the genomic regions (A, -9kb; B, -11.5kb; C, +9kb) from the *Pou3f1* locus showing NANOG binding as determined by ChIP-seq by from Murakami et al. (2016), and PCR genotyping of single embryos (left) showing the wild type and the corresponding deleted bands by gel electrophoresis. 1kb Ladder molecular weight marker is shown on the left of the gels.

Biology Open • Supplementary information

Table S1

Click here to Download Table S1

Table S2

Click here to Download Table S2

Biology Open • Supplementary information
